# Early Detection of Osteoporosis by Dual Energy X-ray Absorptiometry

**DOI:** 10.12669/pjms.306.5566

**Published:** 2014

**Authors:** Asadullah Makhdoom, Makhdoom Rahopoto, Khaleeque Ahmed Siddiqui, Ghulam Ali Qureshi

**Affiliations:** 1 Asadullah Makhdoom, Assistant Professor, Department of Orthopaedic Surgery & Traumatology, Liaquat University of Medical & Health Sciences Jamshoro, Sindh, Pakistan.; 2 Muhammad Qasim Rahopoto, Associate Professor, Department of Medicine, Liaquat University of Medical & Health Sciences Jamshoro, Sindh, Pakistan.; 3 Khaleeque Ahmed Siddiqui, Professor, Department of Orthopaedic Surgery & Traumatology, Liaquat University of Medical & Health Sciences Jamshoro, Sindh, Pakistan.; 4 Ghulam Ali Qureshi, Professor, Medical Research Center, Liaquat University of Medical & Health Sciences Jamshoro, Sindh, Pakistan.

**Keywords:** Osteopenia, Osteoporosis, Dual Energy X-ray Absorptiometry

## Abstract

**Background and Objective:** An early detection of osteoporosis through DEXA procedure will not only improve the disease management practices but also would help in impeding national productivity losses by mass screening and awareness. Our objective was to measure efficacy of DEXA procedure in early detection of osteoporosis and prevention of its complications.

**Methods:** This case series observational study was designed to confirm the bone mineral density by dual energy x-ray absorptiometery (DEXA). The duration of study was three years from November 2010 to October 2013. Subjects aged between 30 (when the risk of osteoporosis is low) to 60 (when osteoporosis is almost sure to be found).

**Results:** Three hundred thirty patients were evaluated. There were 23 (6.96%) male and 307 (93.03%) female. Normal male were 09 (39.10%), osteopenia 11 (47.80%) and osteoporosis 3 (13%). Normal females were 72 (23.50%), osteopenia 140 (45.60%) and osteoporosis 95 (30.90%). P-value was more then 0.005 and not significant. Mean age was 48.73, minimum 30, maximum 60 and SD 7.247. Population category distribution was 243(76.6%) from urban and 87(26.4%) rural. In urban areas normal were 56(23%), osteopenia 113(46.50%) and osteoporosis 74(30.50%). In rural areas normal 25(28.70%), osteopenia 38(43.70%) and osteoporosis 24(27.60%). P-value was 0.567. Out of 330 there were 81(24.54%) normal, 98 ((29.69%) osteoporosis and 151 (45.75%) osteopenia.

**Conclusion:** Osteopenia was the most common diagnosis mostly in younger age group and early diagnosis of this problem can help prevent osteoporosis.

## INTRODUCTION

Osteoporosis was defined by Albright F et al.^1^ in 1941, as a pathological condition in which there is lack of bone tissue, but that tissue which remains is fully calcified’. This definition of osteoporosis is, however, impractical, as it is based on pathology for a condition that has to be identified clinically via radiological assay.

Osteoporosis develops when bone resorption occurs too quickly and replacement occurs too slowly. The trabecular changes produce a loss of trabecular connectivity that is particularly damaging to bone strength.^2^ Early diagnosis of osteoporosis, fracture risk prediction, and assessment of efficacy of therapy therefore are of great interest.^3^

Diagnosis of osteopenia from conventional radiographs also depends on the experience of reader and subjective interpretation.^4^ Therefore, the sensitivity of conventional radiography to detect early bone loss based on increased radioluency is generally considered to be low.^5^ Dual X-ray Absorptiometry DEXA^6^ uses an X-ray tube instead of a radionuclide as a photon source. The dual energy effect is achieved by either alternating k-edge filtration. DEXA, a much more effective clinical tool, decreases the examination time and improves spatial resolution. The time needed for examination of lumbar spine is less than five minutes. The major advantage of DEXA is its ability to measure skeletal density at sites other than the extremities, especially lumbar spine and proximal femur. In particular, significant demineralization was demonstrated by radial DEXA in many patients whose lumbar DEXA measurements were within normal limits.^7^

Osteoporosis is the most common bone disease and affects both men and women. The clinical and public health implications of this disease are considerable because of the mortality, morbidity, and cost of medical care associated with osteoporotic fractures is diagnosed on the basis of a low-impact or fragility fracture or low bone mineral density assessed by central dual-energy x-ray absorptiometry.^8^

There is also controversy regarding the work-up for patients who have been diagnosed as having osteoporosis based on bone mineral density. It is difficult to decide where interventions should be targeted both from a patient's perspective and for cost effectiveness.^9^

The socially and economically vulnerable groups in underdeveloped countries like Pakistan are the worst sufferer of the disease and as disabling complications where most of the time poor victims whether men or women neglect the condition till it gets worse. Due to less awareness and because of financial constraints the victims usually take self-medications and use ordinary analgesics readily available in the market and are advised by one of their relative or friend. They keep on changing the type of analgesic till they reach a condition severely affecting their workability. By this time the management, prognosis and recovery of the patient enters an adverse state and their health and productivity are compromised. Although no organized data is available regarding the impact of osteoporosis on individual and national productivity, it is firmly believed that if estimated scientifically, an alarming situation would certainly be revealed. The objective of this study was to measure efficacy of DEXA procedure in early detection of osteoporosis and prevention of its complication.

## METHODS

The study was conducted at Department of Bone Mineral Density Liaquat University of Medical & Health Sciences Jamshoro from November 2010 to October 2013. It was an observational case series, designed to confirm the bone mineral density of subjects by dual energy x-ray absortiometry (DEXA) Hologic Discovery A.

Subjects aged between 30 years (when the actual risk of osteoporosis is not so common) and 60 years (when osteoporosis is most likely to be found) were included. The study was not gender biased; however, women were focused more because of their vulnerability for this disease, particularly due to hormonal changes at menopause (40-50 years of age). Willing patients were registered at the outpatient and emergency department of Liaquat University Hospital, Jamshoro & Hyderabad. The aims, objectives and the importance of research were read to them before asking for their consent in writing.

The patients having any disease, treatment or any medical problem/procedure capable of influencing the results of the study were excluded. A pre project field-tested proforma was filled and kept in record. Data was entered in SPSS version 21. The WHO defined criteria for diagnosis of osteoporosis & low BMD was used for analysis.^10^

## RESULTS

***Statistical Analysis Procedure: ***Data was analyzed using SPSS version 21.0. Frequency and percentage were calculated for all categorical variables like sex, complication, category of population, occupation and result/outcome/disease and these frequencies and percentages were displayed through graphs. Mean and Standard deviation (SD) were calculated for all quantitative variables like age, hip neck, hip neck Z-score & T-score, hip total, hip total Z-score & T-score, spine, spine Z-score & T-score, calcium, serum phosphate, S. Alkaline Phosphate, Vitamin D. Chi-square test was applied to check significant association between categorical variables gender and disease, occupation and disease, population category and disease, complication and disease.

Three hundred thirty patients were evaluated which included 23 (6.96%) male and 307 (93.03%) female.

Nine (39.10%) males were normal, 11 (47.80%) were osteopenic and 3 (13%) males had osteoporosis. Number of normal females was 72 (23.50%), osteopenic 140 (45.60%) and osteoporosis 95 (30.90%). P-value was more then 0.005 and not significant. Statistical significant association between variables disease /result /outcome and gender was measured by applying Chi-square test.

Mean age was 48.73, minimum 30, maximum 60 and SD 7.247. Mean T-score of hip was -1.3592, median -1.5000, mode -1.90, minimum -1.4, maximum 6.90 and standard deviation was 1.56595. Mean T-score of spine was -1.5043, median -1.6000, mode -1.80, minimum -5.20, maximum 3.70 and standard deviation was 1.38099.

**Table-I:** Statistical significant association between variables Result/Outcome/Disease by age and scanned areas through DEXA.

**Statistics**											
		**Age**	**H. Neck**	**H.N** **T-score**	**H.N** **Z-score**	**H. Total**	**H.T** **T-score**	**H.T** **Z-score**	**Spine**	**Spine** **T-score**	**Spine** **Z-score**
N	Valid	330	326	326	326	328	328	328	325	325	325
	Missing	0	4	4	4	2	2	2	5	5	5
Mean		48.73	.7017	-1.3592	-.5831	.8450	-.7884	-.2909	.8767	-1.5043	-.7972
Median		50.00	.6835	-1.5000	-.7500	.8300	-.9000	-.4000	.8750	-1.6000	-.9000
Mode		55	.78	-1.90	-1.00	.86	-1.30	-.80	.83^a^	-1.80	-1.90^a^
Std. Deviation		7.247	.15578	1.56595	1.32667	.16293	1.29267	1.25964	.14747	1.38099	1.32095
Minimum		30	.14	-14.50	-3.30	.18	-3.80	-3.30	.41	-5.20	-4.00
Maximum		60	1.62	6.90	7.30	1.86	7.50	7.80	1.38	3.70	3.80

a. Multiple modes exist. The smallest value is shown.

Population category distribution was 243(76.6%) from urban and 87(26.4%) rural. In urban areas normal were 56(23%), osteopenia 113(46.50%) and osteoporosis 74(30.50%). In rural areas 25(28.70%) were normal, osteopenic were 38(43.70%) and 24(27.60%) had osteoporosis. P-value was 0.567. Statistical significant association between variables Result/Outcome/disease and Population category was measured by applying Chi-square test.

Patients presented with various complaints like whole body pain 223 (67.57), multiple joint pain 57 (17.27%), back pain 44 (13.3%) and neck pain 6 (1.8%). As regards occupation housewives were 249 (75.5%), teachers 33 (10.0%), doctors 19 (5.8%), laborers 10 (3.0%) and government / private servants, nurses, lady health visitors, advocates engineers were 14 (9.18%). Statistical significant association between variables Result/Outcome/Disease and Occupation was measured by applying Chi-square test.

**Table-II:** Statistical significant association between variables Disease by Occupation

Occupation * Disease Cross tabulation							
			**Result**			**Total**	**P-value**
			**Normal**	**Osteoporosis**	**Osteopenia**		
Occupation	Advocate	n (%)	0 (0%)	1 (100%)	0 (0%)	1 (100%)	**0.295**
	Nurse	n (%)	0 (0%)	1 (33.3%)	2 (66.7%)	3 (100%)	
	Service	n (%)	1 (33.3%)	0 (0%)	2 (66.7%)	3 (100%)	
	Teacher	n (%)	13 (39.4%)	6 (18.2%)	14 (42.4%)	33 (100%)	
	Banker	n (%)	1 (100%)	0 (0%)	0 (0%)	1 (100%)	
	Doctor	n (%)	6 (31.6 %)	4 (21.1%)	9 (47.4%)	19 (100%)	
	Engineer	n (%)	1 (33.3%)	0 (0%)	2 (66.7%)	3 (100%)	
	G. service	n (%)	0 (0%)	0 (0%)	3 (100%)	3 (100%)	
	Labor	n (%)	2 (20%)	4 (40%)	4 (40%)	10 (100%)	
	House Wife	n (%)	56 (22.5%)	79 (31.7%)	114 (45.8%)	249 (100%)	
	LHV	n (%)	0 (0%)	0 (0%)	1 (100%)	1 (100%)	
Total		n (%)	81 (24.5%)	98 (29.7%)	151 (45.8%)	330 (100%0	

Mean calcium was 8.82, median 8.90, mode 8.90, minimum 8.0, maximum 11.70 and standard deviation 0.981. Mean phosphorus was 3.80, median 3.70, mode 4.00, minimum 1.045, maximum 9.80 and standard deviation 1.045. Mean vitamin D was 16.83; median 13.85, mode 5.40, minimum 1.50, maximum 87.90 and standard deviation were 12.20.

**Table-III:** Statistical significant association between Bone Mineral Levels in all involved subjects

**Statistics**						
		**Age**	**Calcium**	**Serum Phosphorus**	**S. Alkaline Phosphorus**	**Vit-D**
N	Valid	330	325	326	329	328
	Missing	0	5	4	1	2
Mean		48.73	8.8295	3.8012	159.8964	16.8394
Median		50.00	8.9000	3.7000	129.0000	13.8500
Mode		55	8.90	4.00	140.00	5.40
Std. Deviation		7.247	.98108	1.04516	137.93238	12.20731
Minimum		30	.80	-3.00	8.30	1.50
Maximum		60	11.70	9.80	1542.00	87.90

Out of 330 there were 81(24.54%) normal, 98 ((29.69%) osteoporosis and 151 45.75%) osteopenia.

## DISCUSSION

In Pakistan there is no clear data available on the number of osteopenia or osteoporosis particularly in young age group and also there is a lack of information on epidemiology and demographics of fractures in their future. Diagnostic tools and facilities for osteoporosis are available and localized to large cities. Twenty-five DEXA machines are available throughout the country and only three are in public sector universities. Our university is the first to purchase & start this diagnostic tool to evaluate and facilitate the population, and about 150 ultrasound machines are also available and mostly promoted by pharmaceutical companies. This clinical based study was designed to ascertain the efficacy of DEXA in early detection of osteoporosis and in prevention of its complications due to late detection.

Osteoporosis is common in elderly patients especially menopausal women aged above 40 years. In a public sector general hospital usually the orthopedic OPD attendance is not less than 100 patients per day. Out of these 100 patients at least 30 to 40(%) are above the age of 40 years, and complain of joint and skeletal pain. When diagnosed radiologically, about 50% of these are osteopenic and 23% are osteoporotic. Normal lateral radiolographs are only able to detect the disease when 20 to 40(%) bone mass are already lost. This severely affects the prognoses of disease and complete recovery because with loss of 40% bone mass it is highly difficult to reverse the process and ease back the patient’s life to normal. The situation requires a more precise method of investigation to assess the mineral composition in osteoporosis patients. Other orthodox tests, if taken as supplementary measure for early detection do not help much because in almost all cases, early disease is not revealed. Hence, affiliated pathological, biochemical and other tests for ascertaining osteoporosis at an early stage are relatively expensive and are very complicated, so results by ordinary labs in this regard cannot be trusted much. In Pakistan population is growing rapidly and the number of aged peoples is increasing continuously, particularly in women osteoporosis is also a remarkable public health problem.^11^^-^^13^

Osteopenia and osteoporosis is associated with older age and over the age of 45 years 16% women were found osteoporosis and also affected one in 6 women up to the age of 50 years as similar to western studies.^14^

Fortunately, parallel methods are available to diagnose the disease at an early stage, so that management can be ensured before tangible complications develop. Bone mineral density test is currently the best available technique by Dual Energy X-ray Absorptiometry (DEXA). This diagnostic test is measuring the whole bone mass density but particularly from three different areas from body like hip, spine and wrist and also non invasive, painless and very easy to perform and taking not more then ten minutes. There is also minimum radiation and risk to the patient is zero percent and providing accurate results.

A cross-sectional study conducted in an obstetrics/gynaecology setting at Quetta, Pakistan, which included 334 women older than 20 years of age were assessed by quantitative ultrasonography to find out the risk factors for osteoporosis. In this study the normal, osteopenic and osteoporotic women were compared and chi-square test applied for categorical variables, 146 (43.7%) women were reported to be normal, 145 (43.4%) were osteopenic and 43 (12.9%) were osteoporotic. The mean age and standard deviation of the participants were 36.7 years +/- 13.0 years.^15^

In another cross-sectional study, conducted through an osteoporosis knowledge assessment questionnaire (OKAT) and data collected by a face-to-face interview about osteoporosis symptoms, risk factors orientation, prevention and treatment. Total 320 healthy women were divided in three groups aged 25-35, 36-45 and over 45 years and concluded that the majority had the modest knowledge regarding osteoporosis and Younger women were found to have low bone mass and premature osteoporosis which is similar to our study findings.^16^

## CONCLUSION

Osteopenia was the most common diagnosis mostly in younger age group. Early diagnosis can prevent development of osteoporosis. The outcome of the study with an ordinary clinical trial done for impact evaluation as an effort to set right the management practices of osteoporosis along with appropriating it’s academic and research configuration.
